# The oxygen carrier M101 alleviates complement activation, which may be beneficial for donor organ preservation

**DOI:** 10.3389/fimmu.2022.1006761

**Published:** 2022-09-12

**Authors:** Bénédicte Puissant-Lubrano, Charlène Bouthemy, Nicolas Congy-Jolivet, Jean Milhes, Vincent Minville, Nassim Kamar, Leïla Demini, Franck Zal, Yves Renaudineau

**Affiliations:** ^1^ Immunology department laboratory, Institut Fédératif de Biologie, Toulouse University Hospital Center, Toulouse, France; ^2^ INFINITy, Toulouse Institute for Infectious and Inflammatory Diseases, INSERM U1291, CNRS U5051, University Toulouse III, Toulouse, France; ^3^ CRCT, INSERM UMR 1037, University Toulouse III, Toulouse, France; ^4^ Department of Anesthesiology and Critical Care, Toulouse University Hospital Center, Toulouse, France; ^5^ Department of Nephrology and Organ Transplantation , Toulouse University Hospital Center, Toulouse, France; ^6^ HEMARINA, Aéropôle Centre, Morlaix, France

**Keywords:** complement, C3 convertase, inhibition, extracellular hemoglobin, M101

## Abstract

During organ transplantation, ischemia/reperfusion injury and pre-formed anti-HLA antibodies are the main cause of delayed graft function and recovery through the activation of the complement system. By supplying oxygen during transplantation, M101 is suspected to avoid complement activation, however, a direct effect exerted by M101 on this pathway is unknown. This was tested by using functional assays (lymphocytotoxic crossmatch test, C3d Luminex-based assay, 50% complement hemolysis [CH50], and 50% alternative complement pathway [AP50/AH50]), and quantitative assays (C3, C3a, C4, C5, C5a, C6, C7, C8, C9 and sC5b-9). M101 interferes with the anti-HLA lymphocytotoxic crossmatch assay, and this effect is complement-dependent as M101 inhibits the classical complement pathway (CH50) in a dose-dependent and stable manner. Such inhibition was independent from a proteolytic effect (fractions C3 to C9) but related to a dose-dependent inhibition of the C3 convertase as demonstrated by exploring downstream the release of the anaphylatoxins (C3a and C5a), C3d, and sC5b-9. The C3 convertase inhibition in the presence of M101 was further demonstrated in the AP50/AH50 assay. In conclusion, the use of M101 avoids the activation of the complement pathway, which constitutes an additional advantage for this extracellular hemoglobin to preserve grafts from ischemia/reperfusion injury and preformed anti-HLA antibodies.

## Introduction

M101 is an extracellular soluble hemoglobin (Hb) derived from the marine invertebrate *Arenicola marina* sharing high homology with adult human hemoglobin HbA and with the particularity of being a giant and hexagonal-bilayer Hb (3600 kDa, 25x15 nm nanoparticle size) as compared to the 64 kDa of the HbA (1, 2). M101 has the ability to fix 156 oxygen molecules (versus 4 for HbA) and controls the oxidative stress through a superoxide dismutase activity based on copper and zinc (3, 4). M101 is used as a clinical device supplemented in preservation solutions, in static cold storage and machine perfusion, in order to improve transplant preservation from an ischemia/reperfusion injury, and with a breakthrough reported regarding graft recovery and long-term survival (5, 6). Indeed, during organ transplantation, the period of ischemia leads to ATP depletion, acidosis, and reactive oxygen species (ROS) production leading to cell death, complement activation and in turn inflammation, and immune activation (7). Then it could be proposed that part of the M101 properties on graft recovery relies on its capacity to interfere with the complement pathway, either indirectly to reduce complement activation by supplying oxygen and antioxidative effects, or either directly as reported with cell-free HbA (8, 9). Accordingly, this raises the question as to whether or not there is interference with M101 on this pathway.

**Figure 1:**
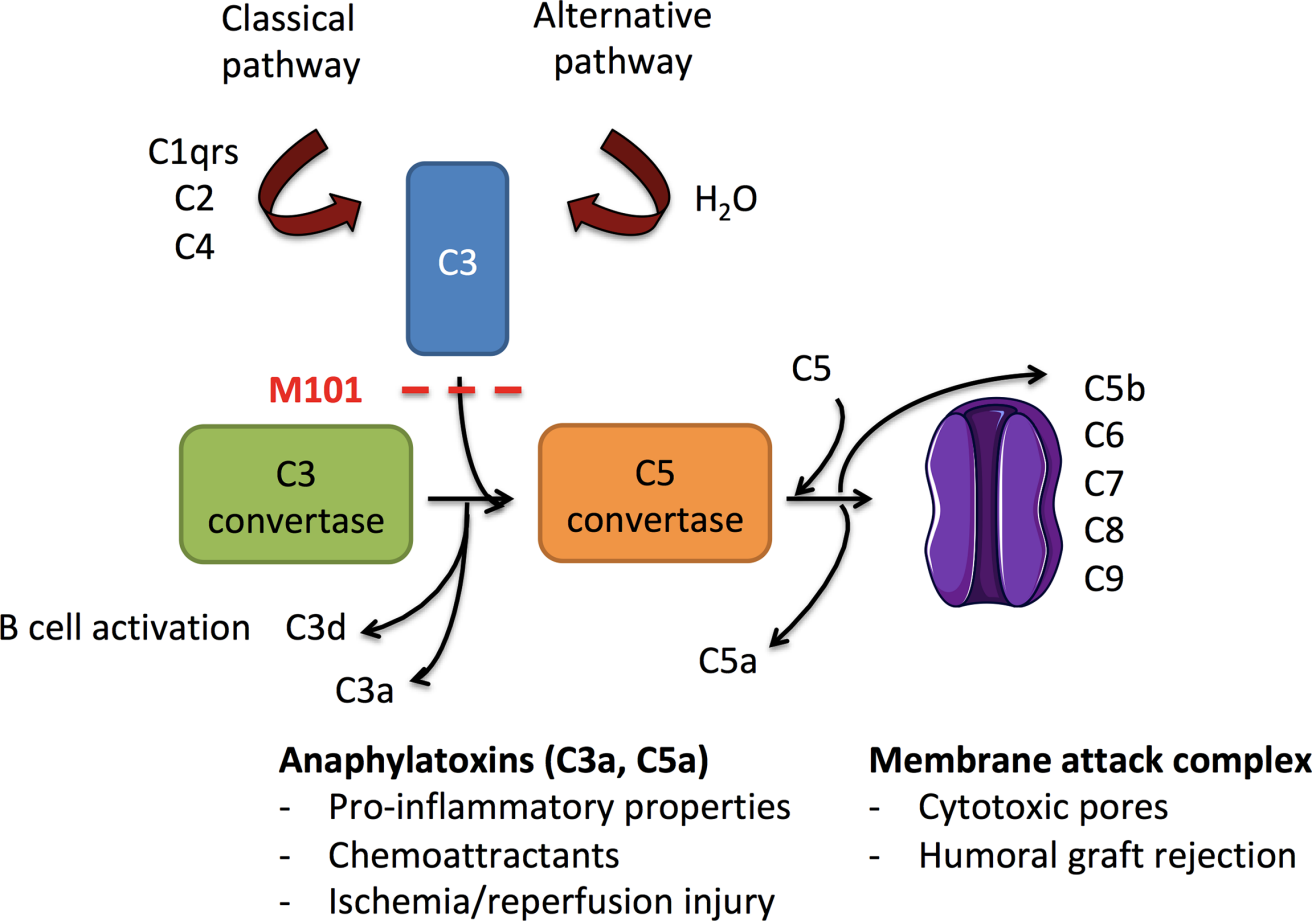
Schematic overview of complement activation in ischemia/reperfusion injury and allograft rejection. Activated following immunoglobulin binding to its antigen (classical pathway) or after the continuous hydrolysis of C3 in an aqueous environment (alternative pathway), both complement pathways lead to the formation of C3 convertases (C4bC2a and C3bBb, respectively). Downstream, C3 convertases cleave C3 into C3a (anaphylatoxin) and C3b. C3b amplifies the complement activation through formation of new C3 convertases and contributes to the formation of C5 convertases (C4bC2aC3b and C3bBbC3b). C3 convertase is further inhibited by factors I and H that finally convert C3b into C3d. C5 convertases cleave C5 into the anaphylatoxin C5a and C5b to generate the membrane attack complex (MAC, C6-C9) and the release of sC5b-9. C3a and C5a possess anaphylatoxin properties, and C3d provides ligands for complement receptors (CR)2 present on B cells. As a consequence, the use of M101 in transplantation may protect from antibody binding (classical pathway), alternative pathway activation, and subsequent effector functions.

The complement pathway, a key element of the innate immune system composed of multiple proteins, needs to be activated in order to contribute to the elimination of invading pathogens, apoptotic or necrotic cells, and immune complexes (8, 9). Complement activation can be initiated by antigen-antibody immune complexes containing immunoglobulin IgG or IgM (classical pathway) leading to the formation of the complement (C)1 complex (C1qrs) to recruit the protease C2 plus C4, the latter two being also activated by the lectin pathway (**Figure 1**). In addition, the spontaneous hydrolysis of the C3 protein in a fluid phase together with factor B and activated factor D, initiates the alternative pathway and leads to the binding of C3b on a foreign surface. Once activated, the three pathways converge to a common pathway at C3 to form the C3 convertases (C4bC2a or C3bBb) and progress to the formation of C5 convertases (C4bC2aC3b or C3bBbC3b) that are necessary for the constitution of the membrane attack complex (MAC, C5b-9) on the target membrane. During C3 and C5 convertase formation, functional activated fragments are generated including the anaphylatoxins C3a and C5a that attract phagocytic cells, and the antigen bound C3d derived from bound C3b that regulates the antibody (Ab) response (10). Accordingly, and from a therapeutic point of view, selective inhibition of the complement pathway has shown promise in transplantation to prevent ischemia/reperfusion injury (alternative and lectin pathways) and allograft antibody mediated rejection (classical pathway) (11).

In this study, we tested and observed an interference of M101 on the complement-dependent cytotoxicity crossmatch (CDC-XM) reaction. To go further, such analysis was completed by exploring the capacity of M101 to affect the classical and alternative pathways using functional (C3d Luminex-based assay, 50% complement hemolysis [CH50], and 50% alternative complement pathway [AP50/AH50]) and quantitative assays (C3, C3a, C4, C5, C5a, C6, C7, C8, C9 and sC5b-9). The main observation from such analysis was that M101 was effective, at concentrations used in the graft preservation solution (1-5 g/L), to control C3 convertase, which may explain in part the better recovery of renal function associated with the use of M101 as an additive to the graft preservation solution (5, 6, 12).

## Material and methods

### Healthy controls

Healthy staff members of the medical laboratory of the University Hospital of Toulouse (CHU de Toulouse, France) and of Brest (CHU Brest, France) were selected. Exclusion criteria, when known, included immune-related diseases, and an active infection in the last 6 months. Peripheral blood was collected in uncoagulated and EDTA-anticoagulated tubes. All individuals were volunteers, have given their informed consent, and the study was conducted in accordance with the Declaration of Helsinki Principles.

### Complement-dependent cytotoxicity crossmatch assay

As previously described with adaptations (13), B and T cells were sorted using an EasySep^®^ Kit (STEMCELL Technologies, Vancouver, Canada). Next, sorted B and T cells were incubated 30min at 22°C with: (i) heat-inactivated AB human serum used as a negative control (Sigma-Aldrich, Saint-Louis, Mo); (ii) heat-inactivated sera from patients containing human IgG/IgM anti-HLA class I and/or II DSA and qualified as lymphocytotoxicity positive controls as covered by the authorization numbers DC20162804 by the French Ethical Southwest and Overseas Committee (SOOM2); (iii) chimeric anti-HLA class-I human monoclonal IgG1 (*In vivo*gen^®^, Toulouse, France) (14); (iv) IgG anti-lymphocyte mAb (One Lambda, Thermo-Fisher, Canoga Park, CA); or (iv) IgM anti-human β2 microglobulin mAb (BD biosciences, San Jose, CA). Rabbit serum containing complement (Servibio, Meudon, France) preincubated up to 8h with increasing amounts of M101 (buffer alone, 1 g/L, and 5 g/L; HEMO_2_life^®^, Hemarina, Morlaix, France) was then added in Terasaki plates (Greiner Bio-One, Les Ulis, France) for 60 min at 22°C. Separation between live (colored in green) and dead cells (colored in red) was achieved by fluorescent microscopy, using the FluoroQuench^®^ staining/quenching reagent (One Lambda). Results were scored as follows: score 1: 1-10% dead cells; score 2: 11-20%; score 4: 21-50%; score 6: 51-80%; and score 8: 81-100%.

### C3d single-HLA antigen bead assay

The Lifecodes^®^ C3d single-antigen Luminex based assay (Immucor, Stamford, CT) was adapted as follows: single antigen beads (40 µL) were incubated 30 min at 22°C with 10 µL of heat-inactivated CDC-XM positive control serum containing anti-HLA class I Ab or from a pool of human anti-HLA class I Ab (Immucor, Ref: LSAPC1). Next, 30 µL of a solution containing human complement fractions (CD3dCS, Immucor) was added. This solution was pre-incubated 1h with 10 µL of increasing amount of M101 (buffer alone, 1 g/L and 5 g/L final concentration). After 30 min at 22°C, and several wash steps, a phycoerythrin coupled anti-C3d mAb was added in order to recognize the C3d fraction bound to the beads in response to the classical complement pathway activation. Finally, the median fluorescence intensity (MFI) for each HLA class-I bead was determined on a Luminex 200 reader (Luminex Corporation, Austin, TX) and analyzed with MatchIT software (Immucor).

### CH50 and AP50/AH50

Fixed volumes of freshly collected EDTA-anticoagulated blood for CH50 or serum derived from freshly uncoagulated blood for AP50/AH50, all from healthy controls, were incubated 30 min, 2h, 6h, 12h, 24h and 48h at room temperature with increasing amounts of M101 (buffer alone, 0.25 g/L, 0.5 g/L, 1 g/L, and 5 g/L final concentration). Plasma and sera were then collected after centrifugation, and frozen immediately at -80°C. The kinetic hemolytic assays for quantification of classical and alternative pathways were previously described (15, 16). Briefly, for CH50, EDTA-plasma was added to IgG sensitized sheep red blood cells in the presence of 15 mmol/L CaCl_2_. For AP50/AH50, serum was added to rabbit erythrocytes, both diluted in a buffer containing 8 mmol/L EGTA and 2 mmol/L MgSO_4_, on microplates to assess a continuous measure of the optical density at 540 nm by means of a spectrophotometer microplate reader (iEMS reader/dispenser; ThermoFisher, Beverly, MA). Complement activity was deduced from the calibration range and expressed as a percentage of the lysis produced with the calibrator. In selected experiments, CH50 supernatants from the 6h dose-response effect of M101 on EDTA-plasma or from 120 µg/mL eculizumab treated EDTA-plasma (Soliris; Alexion Pharmaceuticals, Inc., Boston, MA) were recovered after 7 min on IgG sensitized sheep red blood cells, centrifuged and used immediately or frozen at -80°C until C3a, C5a and sC5b-9 quantification.

### Complement fractions (quantitative assays)

EDTA whole blood from healthy controls was incubated with M101 (buffer, 0.5 g/L, 1 g/L, and 5 g/L) at different time points (12h, 24h, and 48h) and complement fractions were quantified in collected EDTA-plasma by turbidimetry (Cobas 8000, Roche, Meylan, France) for complement fractions C3 and C4, and by radial immunodiffusion (The Binding Site, Birmingham, UK) for the fractions C5 to C9. Results were expressed as g/L for C3 and C4 according to manufacturer’s instructions, and as the diameter of the ring (mm) for C5 to C9.

C3a, C5a and sC5b-9 from healthy controls were quantified from CH50 supernatants (see above) by ELISA (MicroVue PLus EIA, Quidel, distributed by Eurobio-Scientific Les Ullis, France), according to the manufacturer’s instructions.

### Statistical analysis

Continuous data are described as median and interquartile range (IQR) 25^th^-75^th^ percentile for non-parametric analysis and as mean ± standard error of the mean (SEM) for parametric analysis. Differences between groups were analyzed using a paired multiple one-way ANOVA, and the Tukey’s test was used for *post hoc* comparison. Data were analyzed using GraphPad Prism 9.2 (La Jolla, CA), and a p<0.05 considered as significant.

## Results

### M101 inhibits crossmatch positivity

**Figure 2:**
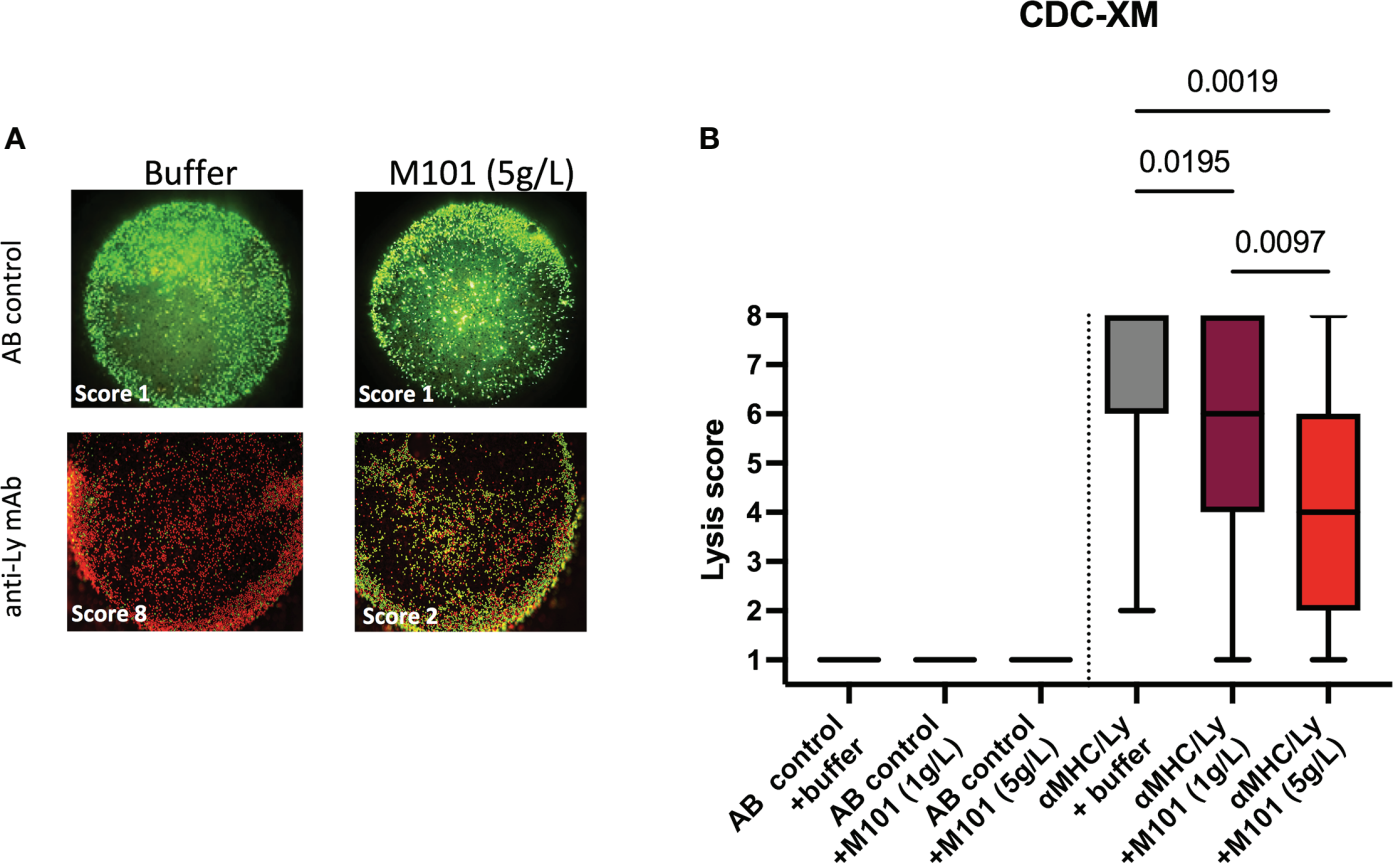
M101 inhibits complement-dependent cytotoxicity crossmatch (CDC-XM) positivity. Sorted B- and T-cells were incubated with a negative AB control serum or a positive control serum (see **Table 1** for details), and next with rabbit complement containing various concentrations of M101 (buffer, 1 g/L, or 5 g/L). Assessment between live (colored in green) and dead cells (colored in red) was achieved by fluorescent microscopy, and a lysis score was established (see material and methods). **(A)** A representative result of T cells incubated with a negative AB control (upstream) or with an anti-lymphocyte (Ly) monoclonal antibody (mAb) used as positive control in the CDC-XM assay (downstream) in the presence of the buffer (left) or M101 at 5 g/L (right). **(B)** Results of 8 independent experiments and results are expressed as box plots of interquartile range. P values are indicated when significant (ANOVA test).

Among patients waiting for transplantation at the university hospital center of Toulouse, up to 34% have pre-formed antibodies to HLA class I/II (CB and NC, personal data) raising the question whether M101, which can be used in organ preservation solutions in transplantation, affects Ab mediated CDC-XM assay using purified T cells (HLA class I) or B cells (HLA class I and II), and rabbit sera as source of complement. Such analysis summarized in **Table 1** and pooled data presented in **Figures 2A, B** revealed that M101 when used at 1 g/L and 5 g/L did not exert a direct cytotoxic effect on T/B cells in the presence of the negative AB human sera control (lysis score =1, **Figure 2B** left). M101 partially affected CDC-XM, in both T and B cells, when used at 1 g/L (median lysis score = 6.0 [IQR: 4.0-8.0] versus 8.0 [6.0-8.0] with buffer; p=0.02) and even more at 5 g/L (median lysis score = 4.0 [2.0-6.0]; p=0.002) (**Figure 2B** right). Altogether, these results support that M101 was effective in reversing at least partially the rabbit complement-dependent cytotoxic effect mediated by the binding of the antibodies in the CDC-XM assay.

**Table 1:** Crossmatch complement dependent lysis score obtained from 8 independent experiments.

Experiment	Abs	Cell type	Buffer	M101 (1 g/L)	M101 (5 g/L)
#1	AB control	T cells	1	1	1
#1	anti-hLy	T cells	8	6	2
#2	AB control	B cells	1	1	1
#2	DSA-I	B cells	8	6	6
#2’	AB control	T cells	1	1	1
#2’	DSA-I	T cells	8	8	6
#3	AB control	B cells	1	1	1
#3	DSA-II	B cells	6	4	4
#4	DSA-I	B cells	8	8	8
#4	anti-hLy	B cells	6	6	4
#5	AB control	B cells	1	1	1
#5	xAb-I	B cells	8	6	4
#6	AB control	B cells	1	1	1
#6	anti-hLy	B cells	8	8	6
#7	AB control	T cells	1	1	1
#7	xAb-I	T cells	6	4	1
#8	AB control	T cells	1	1	1
#8	DSA-I	T cells	2	1	1
#8	anti-hβ2	T cells	8	8	6

B and T cells were purified from peripheral blood human living donors. Abs, antibodies; AB control, AB human serum used as negative control; DSA-I, donor specific Abs anti-HLA class I; xAb-I, chimeric anti-HLA monoclonal antibody targeting anti-human HLA class I public epitopes (500ng/mL); Anti-hβ2, IgM anti-human β2 microglobulin; and anti-hLy, IgG anti-human lymphocytes (target unknown).

### M101 inhibits the classical pathway in a dose-dependent manner

**Figure 3:**
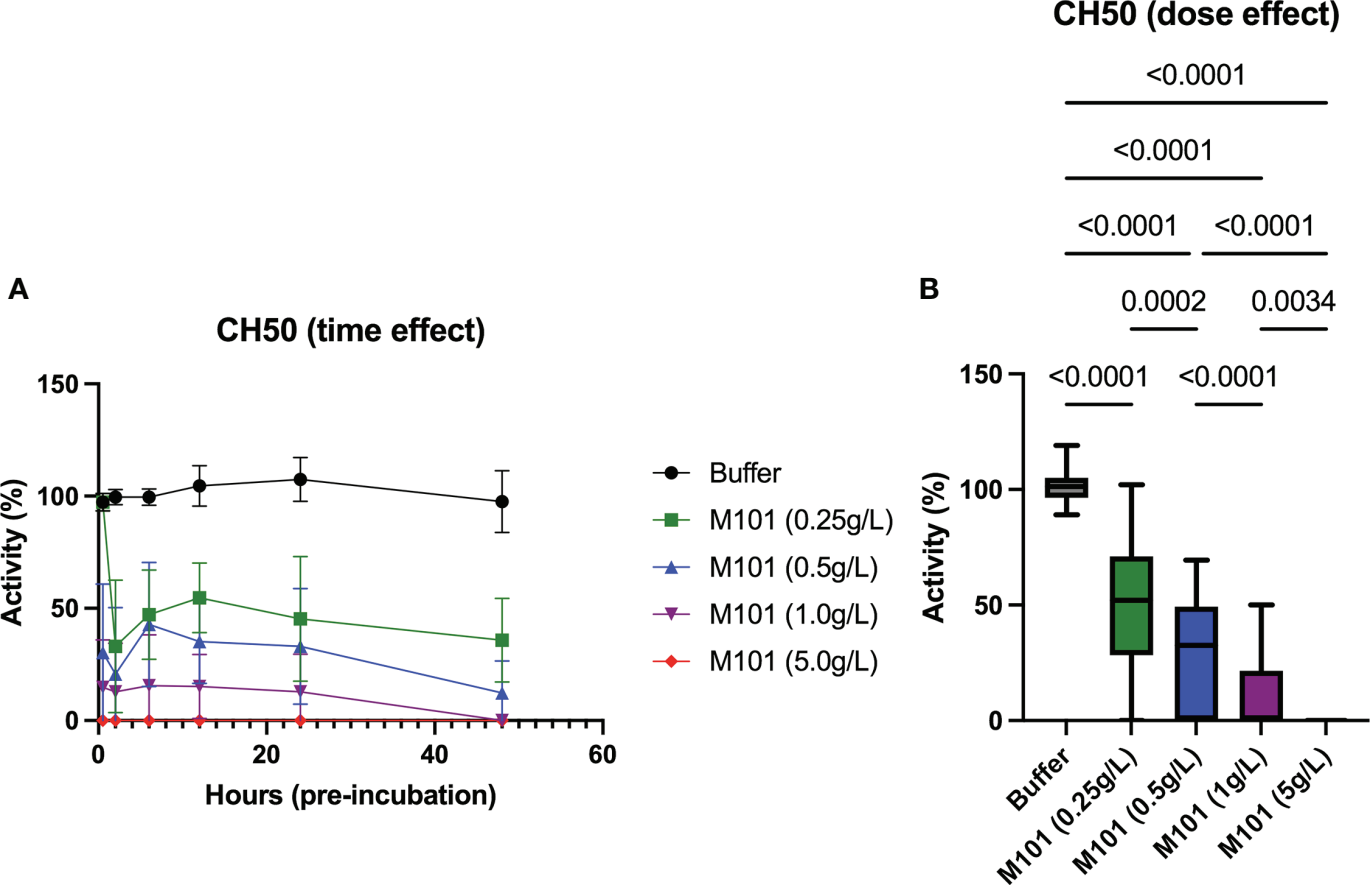
Dose and time-effect of M101 on the 50% classical complement pathway (CH50). EDTA whole blood from 6 healthy controls were pre-incubated with increasing amounts of M101 (buffer, 0.25 g/L, 0.5 g/L, 1 g/L and 5 g/L) during 30 min, 2h, 6h, 12h, 24h and 48h before being centrifuged and EDTA-plasma frozen (-80°C). After that, CH50 activity was evaluated by hemolytic activity using IgG-coated sheep blood cells. **(A)** M101 pre-incubation time effect. **(B)** M101 dose effect, for each concentration all time points are indicated and results are expressed as box plots of interquartile range. P values are indicated when significant (p<0.05).

To go further in the exploration of the M101 capacity to interfere with the CDC-XM, EDTA whole blood from 6 healthy individuals was incubated with increasing amounts of M101 (buffer, 0.25 g/L, 0.5 g/L, 1 g/L and 5 g/L), and then EDTA-plasma was collected at six time points (30 min, 2h, 6h, 12h, 24h, and 48h). Next, the time and dose effect of M101 on the complement classical pathway (CH50) was conducted using these collected plasmas on IgG sensitized sheep erythrocytes. As presented in **Figure 3A**, the M101 capacity to prevent CH50 activation was achieved at the first time point of pre-incubation (30 min) and remained stable through 48h. Compiling results retrieved a dose effect with complete inhibition reported when using M101 at 5 g/L (p<10^-4^), while inhibition of activity was almost complete at 1 g/L (0.0% [IQR: 0.0-21.5%]; p<10^-4^), and partial at 0.5 g/L (32.6% [0.0-49.3%]; p<10^-4^) and 0.25 g/L (52.0% [28.3-71.0%]; p<10^-4^) (**Figure 3B**). Indeed, these findings demonstrated that the activation of the classical complement pathway in the CH50 assay was controlled in a dose-dependent and stable manner by M101.

### M101 does not exert a proteolytic effect on complement fractions

**Figure 4:**
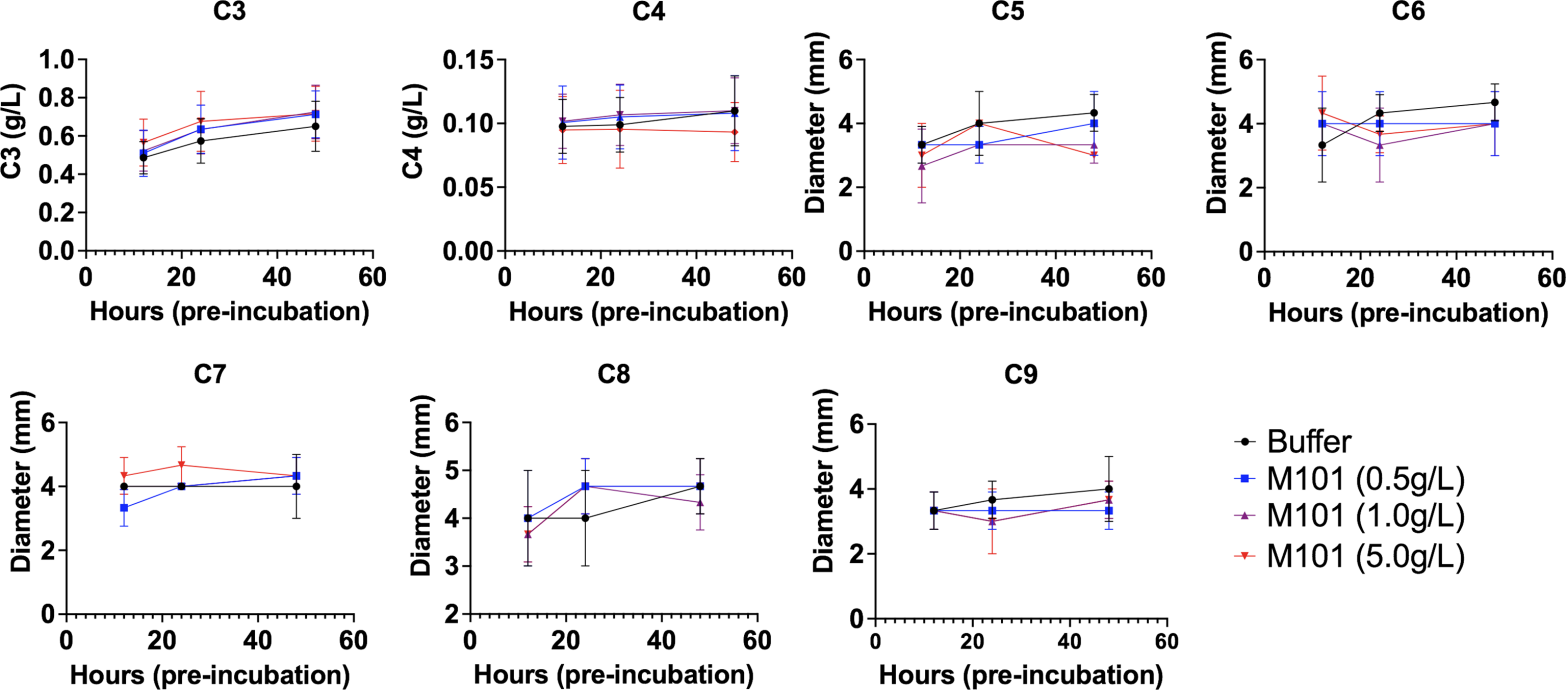
No proteolytic action of M101 on C3 to C9 fractions. EDTA whole blood from healthy controls was pre-incubated with increasing amounts of M101 (buffer, 0.5 g/L, 1 g/L and 5 g/L) during 12h, 24h and 48h before being centrifuged and EDTA-plasma frozen (-80°C). C3 and C4 fractions were quantified by turbidimetry (n=10), results are expressed as g/L, while radial immunodiffusion (n=3) was used for the fractions C5 to C9 and results were expressed as the diameter of the ring (mm). Statistical analysis did not reach significance.

Next and to test the proteolytic effect of M101 on proteins involved in the classical pathway, EDTA whole blood from healthy individuals was pre-incubated with increasing amounts of M101 (buffer, 0.5 g/L, 1 g/L and 5 g/L) and centrifuged at three time points (12h, 24h, and 48h). Next, EDTA-plasma containing complement fractions was evaluated by turbidimetry (C3 and C4; n=10) or radial immunodiffusion (C5, C6, C7, C8, and C9; n=3). As presented in **Figure 4**, C3-C9 fraction levels were stable over time and not affected by addition of increasing amounts of M101. Altogether, these data excluded the possibility that M101 exerted a proteolytic effect on the complement fractions C3-C9. We then postulated that the inhibitory effect of M101 functioned through interaction with the complement macromolecular complexes.

### M101 as an inhibitor of C3 mediated complement activation

**Figure 5:**
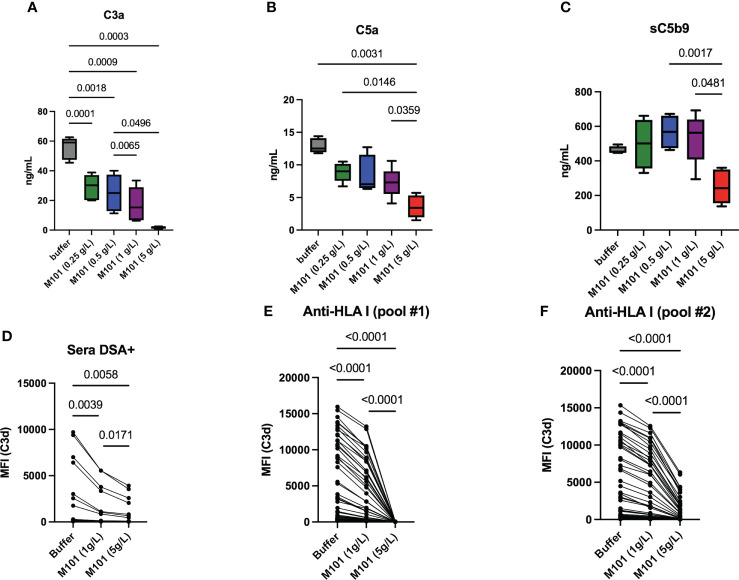
M101 inhibits C3 convertase. **(A–C)** C3 convertase activation and capacity to produce C3a **(A)**, downstream C5a **(B)**, and finally sC5b-9 **(C)** was evaluated by ELISA performed on supernatants collected after CH50 assay. EDTA-plasma (n=5 healthy controls) was obtained from EDTA whole blood pre-incubated 6h with an increasing amount of M101 (buffer, 0.25 g/L, 0.5 g/L, 1 g/L, and 5 g/L). These pre-incubated plasmas were mixed with IgG-coated sheep blood cells in the CH50 assay and supernatants were collected after 7 mns **(D–F)** Capacity to release C3d in a Luminex assay using 96 HLA class-I beads in the presence of M101 (buffer, 1 g/L and 5 g/L), complement fractions, and patient sera containing C3d positive anti-human leukocyte antigen (HLA)-I antibodies from one patient **(D)** or from a pool of patients (**E**: pool #1; **F**: pool #2). Results from the 96 HLA class-I beads are represented. The median fluorescence intensity (MFI) for each HLA class-I specificity is reported. P values are indicated when significant (p<0.05).

Complement common pathway activation involves macromolecular complexes that sequentially cleave C3 to C3a and C3b (C3 convertases), C5 to C5a and C5b (C5 convertases), and contribute to MAC formation with the release of the soluble terminal complement complex (sC5b-9). In order to test these macromolecular complexes, M101-treated plasma selected from 5 healthy donors was incubated with IgG sensitized sheep red blood cells (CH50 assay) and the release of C3a, C5a and sC5b-9 was next quantified in collected supernatants. Compared with the anti-human C5 mAb (Eculizumab) that profoundly inhibited C5a and sC5b-9 generation but had no effect on C3a generation [(15) and data not shown)], M101 significantly inhibited the production of C3a, C5a and sC5b-9 (**Figures 5A–C**). Again, a dose effect was observed with M101 and the maximal effect was retrieved at 5 g/L as this concentration was effective in impairing the formation of C3a (p=0.0003), C5a (p=0.003), and sC5b-9 (p=0.002 *versus* M101 0.5 g/L).

C3 convertase converts C3 in C3a and C3b, and the degradation and inactivation of C3b by the factors I and H lead to the release of C3d. Accordingly, the M101 capacity to inhibit the release of C3d in a Luminex assay with single-HLA class I antigen beads was tested using patient sera containing anti-HLA class-I Abs from one patient or from a pool of patients from the commercial assay, the latter being tested twice (**Figures 5D–F**). M101 was effective in reducing the MFI of anti-C3d staining when used at 1 g/L (p ≤ 0.0004) and 5 g/L (p ≤ 0.006). Of note, the M101 inhibitory effect was not restricted to the anti-HLA Ab target antigen (MFI positive cut-off>1500), but was retrieved for all the 96 HLA class-I antigens (MFI range: 56-15952). Altogether, this suggests an action of M101 on the C3 convertase, which is common with the alternative pathway, or upstream (C1qrs, C2 and C4).

### M101 controls C3 convertase

**Figure 6:**
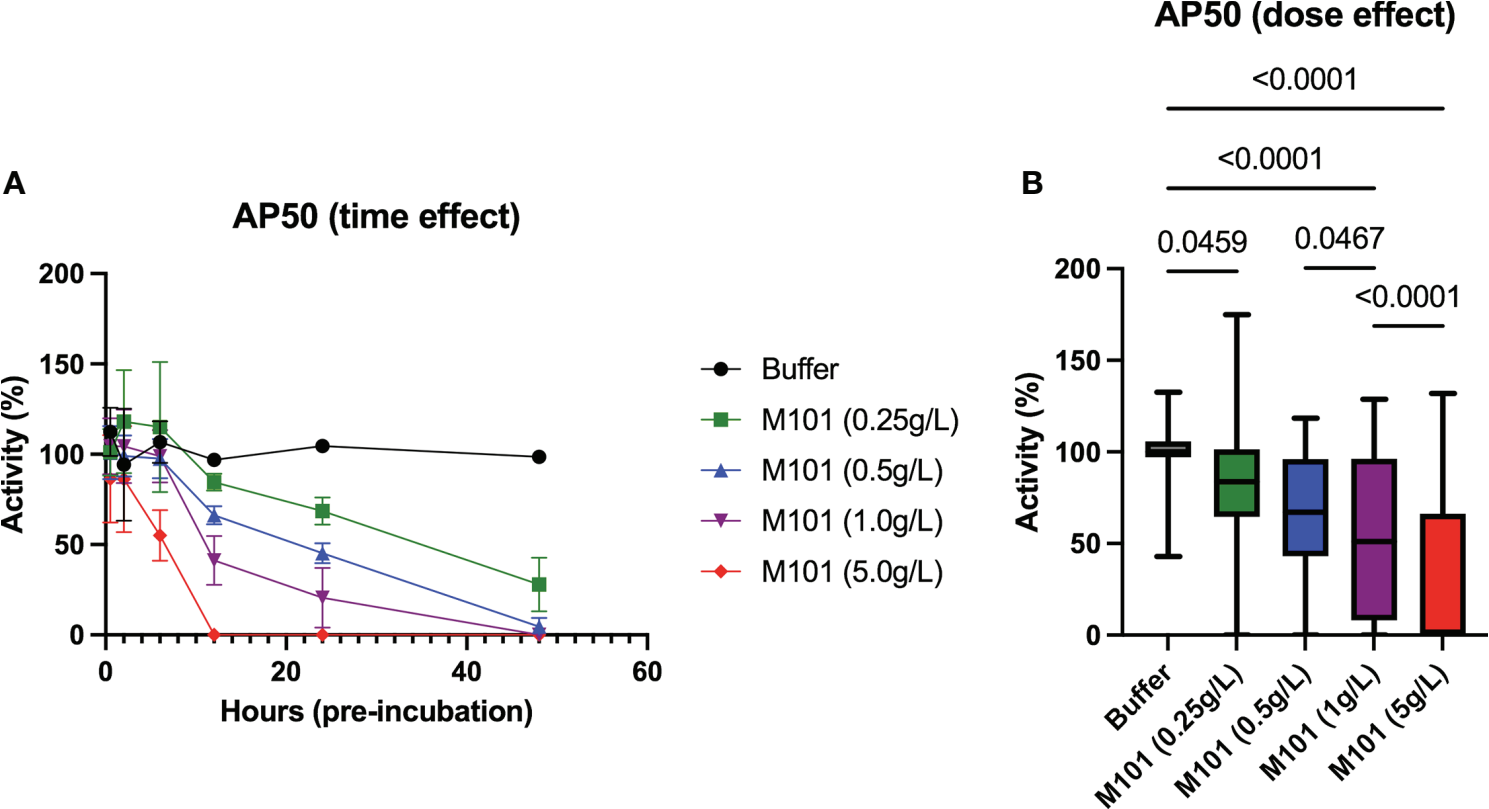
Dose and time-effect of M101 on the alternative complement pathway (AP50/AH50). Whole uncoagulated blood from 5-6 healthy controls were pre-incubated with an increasing amount of M101 (buffer, 0.25 g/L, 0.5 g/L, 1 g/L and 5 g/L) during 30 min, 2h, 6h, 12h, 24h and 48h before being centrifugated and serum frozen (-80°C). AP50 activity was then evaluated by hemolytic activity from sera using rabbit blood cells. **(A)** M101 pre-incubation time effect. **(B)** M101 dose effect, for each concentration all time points are indicated, and results are expressed as box plots of interquartile range. P values are indicated when significant (p<0.05).

Finally, M101 capacity to inhibit the alternative pathway was evaluated as this pathway starts with the formation of the C3 convertase in an amplification loop process independent from Ab mediated C1qrs, C2 and C4 activation. To this end, sera from 5-6 healthy controls were incubated with increasing amounts of M101 (buffer, 0.25 g/L, 0.5 g/L, 1 g/L, and 5 g/L) at different time points (30 min, 2h, 6h, 12h, 24h, and 48h). By using these sera, time and dose effects of M101 were evaluated with the alternative pathway method (AP50/AH50), which evaluated lysis of rabbit erythrocytes in the presence of Mg^2+^. As presented in **Figure 6A**, the maximal inhibitory effect on AP50/AH50 required a pre-incubation of 12 h with M101 when using M101 at 5 g/L, to 48 h when using M101 at 1 g/L and 0.5 g/L, and the inhibitory effect was not achieved after 48h with M101 at 0.25 g/L. When compiling all data (**Figure 6B**), a dose effect was observed with the lower complement activity reported with M101 at 5 g/L (0.0% [IQR: 0.0-66.3%], p<10^-4^ versus buffer) as compared with M101 at 1 g/L (51.0% [8.0-96.3%], p<10^-4^), 0.5 g/L (67.0% [43.0-96.1%], p<10^-4^), and 0.25 g/L (83.7% [64.5-100%], p=0.05). Altogether, this supports the capacity of M101 to control C3 convertase activation and amplification in a dose-dependent manner.

## Conclusion

Complement activation constitutes a cornerstone in transplantation, including in allogeneic antibody mediated rejection through the classical pathway and ischemia/reperfusion injury through the tissue damage capacity to mediate the alternative pathway (17). As a consequence, taming this pathway is of utmost interest and the description of a direct blockade exerted on the complement cascade by M101 (**Figure 1**) coupled with organ preservation, to reduce complement activation, provide an explanation for the shortened time for graft recovery and long-term survival associated with the use of M101 as an oxygen carrier in the organ preservation solution (5, 6).

The deleterious effect on grafts of allogeneic anti-HLA Abs through complement activation is well established, and new therapeutic strategies are needed to prevent/cure this rejection. Indeed, animal models have shown that early blockade of the common pathway is effective in preventing acute antibody-mediated rejection (18, 19), and when used in transplant recipients with preformed DSA the humanized anti-human C5 monoclonal antibody, eculizumab, prevents in part antibody-mediated kidney rejection (20). Similar to eculizumab, M101 when used at therapeutic concentrations (1 g/L) and even more at 5 g/L in *in vitro* assays, was effective in inhibiting the cleavage of C5 to C5a and C5b, the formation of sC5b-9, and complement-mediated lysis in the CH50 and AP50/AH50 assays (15, 21). However, and in addition to eculizumab, M101 inhibits the complement cascade early in the cascade and thereby prevents the cleavage of C3 into C3a and downstream C3d. Moreover, such effect is not human specific as retrieved in the CDC-XM assay, which uses rabbit sera as the source of complement fractions (13). Rabbit and human complement activation present differences, which may explain the partial effect reported in the CDC-XM assay as compared to the clear dose-effect found on CH50 and AP50/AH50 assays as well as on the formation of C3d (22, 23). Of note and not tested in this study, the amino sequence of the C3 ortholog from annelid is 29% identical with its human counterpart (24), supporting a role for M101 to regulate *in vivo* the level of C3 convertase, and in turn complement pathway activation, in *Arenicola marina*.

As shown in a previous clinical trial, M101, when used in preservation solutions in renal transplantation, prevents delayed graft function (DFG), a signature of ischemia/reperfusion injury (4, 5). The role of M101 on ischemia/reperfusion injury is further supported by our observations that M101 was effective in a dose-dependent manner to block the release of the anaphylatoxins C3a and C5a, an inhibition that is critical to prevent ischemia/reperfusion injury as reported within the C3aR/C5aR knock-down model (25). Indeed, anaphylatoxins are potent mediators of inflammation and exert their innate and adaptive functions *via* their cognate receptors present on phagocytic cells (e.g., neutrophils and macrophages) and T/B cells. M101 also leads to a decrease in C3d deposition from anti-HLA DSA as demonstrated when using a graft immunocomplex capture fluorescence analysis technique. Altogether these results indicate that M101 allows the preservation of allograft organs from complement-mediated injury, which can be achieved by two ways: one indirect through maintaining the aerobic metabolism therefore avoiding the activation of the complement, and one direct through a direct inhibition exerted on the complement pathway. These results bring an explanation of the benefits brought by M101 not only to preserve graft quality but also to avoid inflammation and transplant rejection, which may prevent subsequent use of complement pathway inhibitors such as eculizumab. To go further with the beneficial effect of M101, complement split products (e.g. C3a and C5a) as well as defining a transcriptomic signature should be performed in patients receiving M101-preserved organs versus those receiving not preserved organs.

C3-targeted inhibitors and their transfer to the clinic are considered as promising (26). Indeed, C3 specific inhibitors using peptides and nanobodies platforms have been developed and clinical trials conducted in a large panel of diseases including periodontal inflammation and COVID-19 with AMY-101 (27, 28), in paroxysmal nocturnal haemoglobinuria with pegcetacoplan (29), and in acute antibody-mediated graft injury and age-related macular degeneration with Cp40 (30, 31). In addition to its anti-complement properties, M101 has original characteristics including oxygen carrier, anti-inflammatory, anti-bacterial, and superoxide dismutase antioxidant properties (32, 33). It is further notable that no immunological, allergic or prothrombotic effects were associated with M101 injection, and, in contrast to cell-free HbA, M101 did not lead to hypertension, myocardial infarction, renal damage, complement activation or tissue toxicity, which supports systemic usage (3, 8, 9, 34, 35). All these properties open new perspectives for M101 to treat ischemia pathologies inducing hypoxia, production of ROS, complement activation and inflammation.

In conclusion, we have presented here that M101 inhibits the CDC-XM, classical and alternative complement pathways, and anaphylatoxin C3a and C5a release. Such effects most probably rely on the capacity of M101 to avoid activation of the complement system when used at therapeutic concentrations (1-5 g/L). Importantly, these effects coupled with the exceptional properties of M101 (e.g., anti-inflammatory, antioxidant, non-immunological) open new therapeutic perspectives including in transplantation to prevent ischemia/reperfusion injury and allogenic humoral rejection.

## Data availability statement

The raw data supporting the conclusions of this article will be made available by the authors, without undue reservation.

## Ethics statement

The studies involving human participants were reviewed and approved by DC20162804 by the French Ethical Southwest and Overseas Committee (SOOM2). Written informed consent for participation was not required for this study in accordance with the national legislation and the institutional requirements.

## Author contributions

BP-L and YR have contributed to the conceptualization, methodology design, formal analysis, and original draft writing. BPL, CB, NC-J, JM, and YR: investigation and validation. VM, NK, LD and FZ: provision of resources. All authors contributed to the article and approved the submitted version.

## Funding

HEMARINA and The Binding Site provided reagents to complete this study.

## Acknowledgments

We are thankful to Dr. Wesley H. Brooks (University of South Florida, USA) and Gisèle Touzanne for editorial assistance, and to Dr Boutahar Bendaoud, Camille Taurus, Sylvie Le Nuz, and HLA technicians for technical help.

## Conflict of interest

FZ is founder of HEMARINA and holds stock in the company that produces HEMO2life® (M101). LD is employed by HEMARINA. The authors declare that this study received funding from HEMARINA and The Binding Site. The funder had the following involvement with the study: provided reagents to complete this study.

The remaining authors declare that the research was conducted in the absence of any commercial or financial relationships that could be construed as a potential conflict of interest.

## Publisher’s note

All claims expressed in this article are solely those of the authors and do not necessarily represent those of their affiliated organizations, or those of the publisher, the editors and the reviewers. Any product that may be evaluated in this article, or claim that may be made by its manufacturer, is not guaranteed or endorsed by the publisher.

## Abbreviations

Ab, antibody; AP50/AH50, 50% alternative complement pathway; CDC-XM, complement-dependent cytoxicity crossmatch; CH50, 50% complement hemolysis; DSA, donor specific Ab; EDTA, ethylene-diamine-tetraacetic acid; HLA, human leukocyte antigen; HbA, adult human hemoglobin; IQR, interquartile range; MAC, membrane attack complex; MFI, median fluorescence intensity; sC5b-9, soluble terminal complement complex; SEM, standard error of the mean.
